# Bell's palsy after Sputnik V COVID‐19 (Gam‐COVID‐Vac) vaccination

**DOI:** 10.1002/ccr3.5468

**Published:** 2022-02-23

**Authors:** Omid Mirmosayyeb, Mahdi Barzegar, Mina Rezaei, Negar Baharlouie, Vahid Shaygannejad

**Affiliations:** ^1^ 48455 Department of Neurology School of Medicine Isfahan University of Medical Sciences Isfahan Iran; ^2^ 48455 Isfahan Neurosciences Research Center Isfahan University of Medical Sciences Isfahan Iran; ^3^ 48455 Dental Student Research Center School of Dentistry Isfahan University of Medical Sciences Isfahan Iran

**Keywords:** Bell's palsy, COVID‐19, COVID‐19 vaccination, Sputnik V

## Abstract

A possible association between Bell's palsy and COVID‐19 vaccination has been suggested previously. Here, we report two cases of facial nerve hemiparalysis following the Sputnik V COVID‐19 vaccination in a 27‐year‐old female patient and a 58‐year‐old male patient who were both clinically diagnosed with Bell's palsy.

## Graphical abstract

COVID‐19 vaccination could be associated with many side effects. This case report suggests a possible association between Bell's palsy and COVID‐19 vaccination by Sputnik V COVID‐19 vaccination.

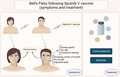

## 1 INTRODUCTION

During the phase 3 of clinical trials of mRNA COVID‐19 vaccinations, seven cases of Bell's palsy in the vaccinated group were reported in both trials combined.^1^, ^2^ Therefore, the US Food and Drug Administration recommended monitoring vaccine recipients for facial paralysis‐related events. Based on the World Health Organization pharmacovigilance database until March 2021, 844 facial paralysis‐related events were identified; including 683 cases of facial paralysis, 168 cases of facial paresis, 25 cases of facial spasms, and 13 cases of facial nerve disorders.^3^

Bell's palsy is caused by the acute weakness of peripheral facial nerve with no identifiable reason and results in unilateral facial paralysis.^4^ Bell's palsy following COVID‐19 vaccination has been previously reported in the scientific literature occurring within days after receiving the Pfizer BioNTech vaccine.^5^, ^6^ However, there are no reports of facial paralysis in recipients of the Russian Sputnik V vaccine, and the results of an interim analysis of a randomized controlled phase 3 trial in Russia did not report any significant adverse events associated with vaccination. Here, we report two cases of Bell's palsy after the first injection of Sputnik V (Gam‐COVID‐Vac) vaccination.^7^

## 2 CASE PRESENTATION

The first patient was an otherwise healthy 27‐year‐old Iranian non‐pregnant female dentist who received the first dose of Russian Sputnik V (Gam‐COVID‐Vac) COVID‐19 vaccination. For the first 2 days following the vaccination, she mainly complained of pain in the injection site, which was followed by fatigue, weakness and low‐grade fever. On the third day, she noticed that the left side of her tongue had become numb, but her taste sensation was intact. Five days after the vaccination, she was not able to close her left eye completely, and she noticed that the left side of her upper lip shifted to the right side while chewing, laughing, or speaking. A day later, the same exact thing happened to her lower lip, and she was unable to drink with a straw. Three hours later, she felt a severe sharp pain along sternocleidomastoid muscle with an ipsilateral radiation to ear, mastoid, and retromaxillary region. Twelve hours later, she was examined by a neurologist. On physical and neurological examinations, she had hemiparalysis of the facial nerve. Her cerebellar examination was normal. She did not have any cutaneous signs of herpes zoster infection. Her vital signs were within normal range, and she was afebrile. She had no history of COVID‐19 infection, and she did not take any medications. Brain MRI was normal. Based on the clinical findings, she was diagnosed with Bell's palsy and treatment was started with corticosteroid (prednisolone with an initial dose of 50 mg per day (1 mg/kg) for one week, which was tapered within 2 weeks) and antiviral agent (virabex, valacyclovir 1000 mg twice a day) within 72 hours after the patient started to show signs. After 10 days, systemic symptoms and facial immobility had resolved, but she was still experiencing a mild pulsating pain in the sternocleidomastoid region.

Patient 2 was a 58‐year‐old Iranian male with a past medical history of controlled diabetes mellitus, presented to the clinic complaining of left facial sudden weakness and difficulty in closing his left eye ten days after receiving the first dose of Sputnik V COVID‐19 vaccine. He received the first injection of the Sputnik V COVID‐19 vaccine, and the following three days, he suffered from hyperthermia, myalgia, and pain in the injection site. Six days later, the patient developed left‐sided facial muscle weakness and reported a tingling sensation on the tongue and lips. One day later, he complained of the left‐sided mouth droop, drooling, decreased taste sensation, slurring of speech, tearing, inability to chew correctly, fully close left eye, smile, and move the left eyebrow. Nine days after the vaccination, he visited our outpatient clinic. Physical examination by a neurologist revealed paresis of his left facial muscles and inability to close his left eye completely. He had no other neurologic deficits. His vital signs were within normal range. There was no history of trauma, skin rashes, and preceding infection. The patient was clinically diagnosed with Bell's palsy, and treatment was started with prednisolone 50 mg (for a week and was tapered within two weeks) and valacyclovir 1000 mg twice a day. At follow‐up in 1 week, his symptoms had partially improved. Further investigations revealed that he had not developed any new signs and symptoms, and he was fully recovered.

## 3 DISCUSSION

The association of Bell's palsy and other viral vaccinations has been discussed previously. A strong association was found between the incidence of Bell's palsy in intranasal inactivated influenza vaccine as well as the influenza H1N1 monovalent pandemic vaccination and parenteral seasonal influenza vaccination.^8^, ^9^, ^10^ A disproportionality analysis of facial paralysis following influenza vaccine showed that the likelihood of reporting facial paralysis following influenza vaccination is higher compared with other vaccines.^11^ The precise mechanism of this association is not yet fully understood. There are hypothetical thoughts proposing that this association might be due to induced response (e.g., reactivation of a herpes virus infection) or autoimmune phenomenon which is thought to occur via either mimicry of host molecules by the vaccinal antigen or the bystander activation of dormant auto‐reactive T cells.^12^ Recent studies show that the risk of facial paralysis in COVID‐19 vaccination is not higher than other vaccines,^3^ and if there is an association between mRNA COVID‐19 vaccines and facial paralysis, it might happen due to the innate immune activation from a combined effect of mRNA and lipids which results in interferon production.^12^ Sputnik V vaccine, however, takes a different route to protection; it is a recombinant vector‐based vaccine that uses adenovirus 26 (Ad26) and adenovirus 5 (Ad5) as vectors to enter cells and express the spike proteins of SARS‐CoV‐2 to trigger the innate immune sensors sufficiently.^13^ To our knowledge, this is the first case report of Bell's palsy after Sputnik V vaccine. Although an association between Bell's palsy and Sputnik V vaccination cannot be established based on a single report, the timing and mode of onset of the Bell's palsy in this patient strongly suggested that it was related to vaccination. We recommend the surveillance of Sputnik V vaccine recipients for any facial paralysis‐related events as the number of injected doses is increasing.

## 4 CONCLUSION

Bell's palsy is a possible side effect of COVID‐19 vaccination and healthcare providers should keep it in mind. More studies are needed to investigate the possible association between COVID‐19 vaccination and Bell's palsy.

## CONFLICT OF INTEREST

All authors declare no conflict of interest relevant to study.

## AUTHOR CONTRIBUTIONS

All the authors listed in the manuscript have participated actively in preparing the final version of this case report.

## ETHICAL APPROVAL

This manuscript was approved by the bioethics committee of Isfahan University of Medical Sciences.

## CONSENT

Written informed consent was taken from the patients to publish the case report.

## Data Availability

No datasets were analyzed or generated during this case report.
